# Childhood narratives and the lived experiences of Hispanic and Latinx college students with uncertain immigration statuses in North Carolina

**DOI:** 10.1080/17482631.2020.1822620

**Published:** 2020-12-09

**Authors:** Carmen Monico, David Duncan

**Affiliations:** a Human Service Studies, North Carolina Agricultural and Technical State University, Greensboro, NC, USA; b Law, Duke University, Durham, NC, USA

**Keywords:** Deportation threat, child immigrant, DACA recipients, health impact, trauma and grief, ambiguous loss, education access, risk and protective factors, legal uncertainty

## Abstract

Childhood narratives are essential to identifying critical issues in their development and developing strategies to reduce risk and build resilience. Ambiguous loss, a psychological risk factor, has emerged as a critical issue among families with uncertain immigration statuses including the recipients of the Deferred Action for Childhood Arrivals program (DACA). This population encounters barriers to accessing mental health resources and educational services, which compel them to use coping strategies to rebound from adversity. Interviews were conducted with 13 students, most being DACA recipients, of Hispanic and Latinx identities attending higher education institutions in North Carolina. The article focuses on the participants’ experience as children prior to the U.S. government decision to rescind their deferred status. Identified risk factors include structural barriers to educational success, health and well-being, and criminalization of undocumented immigrants, confounded with childhood trauma of migration and other mental health consequences. The study found that participants experienced emotional trauma since their arrival in the U.S., starting in childhood, and in some cases, manifesting maladaptive behaviours adversely affecting their health and well-being through their lifespan. The authors recommend comprehensive assistance interventions and more appropriate institutional and community immigrant support systems for these students and their families.

## Introduction

After the USA (U.S.) Congress failed for decades to pass the Development, Relief, and Education for Alien Minors (DREAM) Act, President Barack Obama enacted the Deferred Action for Childhood Arrivals program (DACA) by Executive Branch memorandum on 15 June 2012, to provide relief from deportation to certain undocumented individuals who came to the U.S. as children and grant work permits to eligible adolescents (10–19 years of age) and youth (15 to 24). The Migration Policy Institute estimated that as of 2016, 1.9 million people were potentially eligible for this renewable 2-year period deferred action from deportation and work permits; since then, nearly 900,000 applications have been considered and about 750,000 have been accepted (Gelatt, 2017).

As DACA beneficiaries embrace this temporary status, they settle into another form of uncertainty: one characterized by prolonged waits for program extension and hope for a path to citizenship. In fact, on 5 September 2017, the DACA executive order was rescinded under the Trump administration and as of May 2019, new applications are not being accepted. However, in early 2018, a federal court order renewal allowed this status to be resumed (U.S. Citizenship and Immigration Services (USCIS), 2018) and lower courts have upheld it. Despite the implementation of this executive order, subsequent court orders upholding it, current litigations to extend its renewal, and ongoing Congressional debate on a Dream Act, children of migrant families, particularly those undocumented or without an immigration status, encounter a number of barriers in attaining K-12 and postsecondary education (Colotl, 2017). These barriers undermine their physical, emotional and mental health, as both education and health are essential for child well-being in general and for migrant children in particular.

The two-prompt questions guiding the research were: What is the threat of deportation that Hispanic immigrant college students and their families experience prior, during, and after the decision to rescind the DACA program? What are the health, educational, and socioeconomic impacts this threat has on them and their well-being? The sample was comprised of 13 participants (11 DACA beneficiaries and 2 undocumented immigrants) attending institutions in North Carolina, many of whom are DACA recipients, and the interviews were conducted between September 2017 to August 2018. Among others, the authors analysed the experience of research participants as children, before they turned 18 years old or before entering college, whichever came first. The findings reported in this article focus on how the fear of deportation affected these young adults and their families while growing up, whether they knew of their immigration status during childhood or not, before they and their parents faced the current shift in immigration policies and law enforcement in the U.S. We identified risk and protective factors, and investigated the coping mechanisms the participants use to determine key determinants of resiliency, or the ability to rebound from adversity, as well as the effects of immigration policies on their ability to achieve educational goals through the lens of health and overall well-being.

## Literature review

The participants in this study (adult students attending North Carolina colleges and universities) possessed both protected and unprotected legal statuses: 11 of them were DACAmented and 2 were undocumented immigrants. Given the sample and without intending to conflate the Hispanic or Latinx identity with undocumentation, this article’s literature review focuses on the realities of the continium from legality to illegality in the life trajectory among undocumented and DACAmented youth of Hispanic or Latinx heritage. This review describes the many challenges immigrant children and their families experience in the U.S. Risk factors include structural barriers to educational success due to their fluctuating legal or uncertain statuses and the criminalization of undocumented immigrants resulting from the institutional and structural violence they experience. These are confounded with childhood trauma of migration, living in an undocumented or mixed-status household, and other mental health consequences. Protective factors and coping mechanisms are present at the individual level, within family, in school, and in the community.

### Undocument immigrant transition from protected childhood into an adulthood of illegality: The U.S. experience

Past research suggests that understanding the contexts in which children, adolescents, and youth move from childhood into adulthood, the mediating mechanisms of that environment, and corresponding evaluations of intervention programs must take into account the voices of children when available (Kristén et al., 2015). Research on adolescent development among undocumented young adults confirms that immigrants have more freedom in childhood, to explore, immerse themselves in their surroundings, and to develop greater attachments in schools and neighbourhoods (Gonzales & Vargas, 2015). However, as they grow into young adults, they realize that the legal and community contexts they interact with shape their experiences of “inclusion and exclusion and their feelings of belonging” (Gonzales & Vargas, 2015, p. 61). Throughout their transition from childhoood to adulthood, undocumented children “move from protected to unprotected, from inclusion to exclusion, from de facto legal to illegal” (Gonzales, 2011, p. 602). Undocumented students often progress through the primary school system without experiencing significant differences with respect to their documented peers in their school enviroments given the protective legal policies towards all children, regardless of their status (Abrego, 2006). Nevertheless, upon entering high school, many experience limited higher education opportunities; this “knowledge of future barriers to college attendance leads to a decline in educational motivation” (Abrego, 2006, p. 212).

The DACA program attempted to change that narrative and opened a number of previously inaccessible work and education opportunities for undocumented youth. A national survey of 2,381 “DACAmented” students discovered that the implementation of the DACA program provided “access to new jobs, higher earnings, driver’s licenses, health care, and banking … [and] reduced some of the challenges that undocumented young adults must overcome to achieve economic and social incorporation” (Gonzales et al., 2014, p. 1852). In a study involving 110 immigrant children and 91 parents of Mexican origin, Debry (2012) found that the issue of legality has a more severe impact on children, who are situated at the base of the “injury pyramid,” given the burden they experience due to the fear of deportation, and the imminent threat of family dissolution (p. 829). In fact, there is a growing body of research suggesting that unauthorized status not only affects immigrant adults, but it blocks “progress towards a range of positive youth development outcomes for children and youth” (Yoshikawa et al., 2016, p. 4).

Some of the mechanisms adversely influencing youth development among unauthorized individuals include contextual mechanisms, such as poverty and work conditions, limited access to programs supporting child development, barriers to higher education, and family removal (deportation) proceedings (Yoshikawa et al., 2016). Psychological mechanisms include damaging political rhetoric and social identity threats that undermine their mental health and wellbeing (Yoshikawa et al., 2016). Undocumented youth, particularly the 1.5-generation, find themselves with a complicated transition into adulthood, characterized by pivotal moments compelling them to separate from past routines and adapt to a new reality of “illegality” as part of their life trajectory (Gonzales & Vargas, 2015). Abrego et al. (2017) refers to the “legal violence” undocumented immigrants endure including “physical injury [from] intentional acts to cause harm … [as well as] less visible sources of violence that reside in institutions and structures and without identifiable perpetrators or incidents to be tabulated” (p. 716). In reality, there are numerous systemic barriers immigrant children, adolescents, youth and young adults and their families face, regardless of their legal status.

### Institutional and structural barriers to education among immigrant children, adolescents and youth

An estimated 1.5 million young adults entered the U.S. during their childhood and approximately 4.5 million are children of undocumented parents (Passel & Cohn, 2010), with millions more living in some type of mixed-status home with undocumented family members (Passel, 2006). Of the 1.1. million foreign-born children of unauthorized immigrant parents residing in the U.S. (Passel & Taylor, 2010), about 65,000 graduate high school every year and only 5–10% go on to attend college (Ibarra & Sherman, 2012). The U.S. Supreme Court decision *Phyler v. Doe, No. 80-1538Justia 457 U.S. 202 (1982)* upheld the Equal Protection Clause of the Fourteenth Amendment, granting access to public education for undocumented immigrants and protecting migrant children from mistreatment and forced disclosure of immigration status while in grades K-12. This decision left the question of their access to higher education to the interpretation by each state (Biswas, 2005). In spite of this decision, the effectiveness of child education and the ability to fully utilize its resources are rare.

Overall, immigrant families navigating the U.S. education system find the experience stressful, but research shows that undocumented parents, more than documented parents, are at further disadvantage, complicating the impediments to education these children are forced to surmount (Goździak, 2014). Knowledge and experience within the U.S. higher education system is a luxury many undocumented students do not have as many must complete and submit forms for college applications as first-generation college students (Goździak, 2014). The 1966 report Equality of Educational Opportunity (Coleman) report, commissioned by the U.S. Office of Education in accordance with the Civil Rights Act of 1964, found that having better-educated parents who are on the pathway to citizenship is an important factor in education success and is integral in helping immigrant youth attain high school diplomas (Goździak, 2014). Nevertheless, any educational degree can determine success later in life, especially for immigrant children, adolescents, and youth.

A cross-sectional study of 20 Latinos students of undocumented status in Chicago (Rivera, 2016) identified academic risk factors such as possessing an immigrant status, being a DACA applicant, having to work to pay school, and balancing work and school. For the most part (16) understood the implications of being an immigrant but only 6 of them indicated that they knew of their status since arrival. About half (11) indicated that an immigrant status limits their college choices, 13 of them have disclosed their status, and a similar number reported having been asked for social security number. In this study, assets and resources were considered as protective factors for education. The majority of the study participants identified social responsibility and hope determination as assets. Social responsibility included being part of a collective group, developing service involvement and extracurricular activities and having family responsibility. Nine participants cited DACA benefits as a key resource but more (13) appreciated scholarships and academic awards, and all of them (20) indicated having support from mentors in the form of financial assistance, tuition, employment, school connections, encouragement and advice (Rivera, 2016).

In the face of adversity, children develop maladaptive coping strategies that negatively affect their school performance, lowering standardized test scores on maths and reading in elementary school (Perreira & Ornelas, 2011). The constant fear of deportation, whether parental or personal, that children feel while attending school can disrupt their focus and concentration, making it difficult to both receive and retain information (Perreira & Ornelas, 2011). Another factor determining success in the classroom is the age when a child arrives in the country. Psychologists have found that, before the age of 13 children can learn new languages faster and easier than after that age and the same is true for immigrant children (Hernández et al., 2010). Children who enter the U.S. at a younger age have an easier time acculturating and preparing for an education than an older child, with most over the age of 14 never even entering a classroom (Goździak, 2014).

Most parents active in the education system know the importance of early childhood education on a child’s development; however, many immigrant parents may not know or have access to the necessary resources to provide this education to their children (Mapp & Hornung, 2016). In fact, children aged 0–3 may experience cognitive delays due to economic difficulties, stress, and insufficient formal childcare (Yoshikawa, 2012). Especially among Hispanic children, these resources to early childcare are often underutilized because of their parents’ job responsibilities and cultural reliance on peer and kinship childcare (Yoshikawa, 2012). Thus, undocumented parents encounter many difficulties in providing the right environment for their children to succeed, even if many still believe that simply coming to the U.S. will be enough to provide a bright future for their family.

The complications of their citizenship status often present legal and societal ramifications for themselves and their families. The U.S. Department of Homeland Security Immigration and Customs Enforcement (ICE) has detained and eventually deported parents while picking up or dropping off their children at school, forcing many parents to keep their children from attending school during times of high stress (Mapp & Hornung, 2016). Parental citizenship status can negatively affect the academic achievement of middle school children so that they lose more than a year of formal schooling (Mapp & Hornung, 2016). If the school does not have the resources or the ability to invest in the children it serves, whether through ESL class, additional tutoring, or parental guidance, children are much more likely to suffer and underachieve as a result (Viramontez Anguiano & Lopez, 2012). As a result, the school’s social environment can benefit or undermine the well-being of immigrant children.

Working in tandem with the current political climate and their traumatic histories, research identified poverty to be influential in determining how immigrants experience the education system. Immigrant parents are often forced to work in very poor working conditions, which research has associated with poorer academic success in middle and high school (Suárez-Orozco et al., 2011). These poor conditions produce poor wages that create food and housing insecurity for their families and cause them to invite renters or family to live with them to help pay for the expenses. The crowded house now takes away study space for the children and increases the children’s need to take on home responsibilities, all affecting school success (Suárez-Orozco et al., 2011) and influencing other areas of their well-being. Thus, the impact of poverty is multi-systemic and inter-generational as it affects numerous dimensions and it impacts several generations.

Directly relevant to this study are the immigration policies and social climate in the South, which place additional restrictions on the experiences of undocumented immigrants living in that part of the U.S. In North Carolina, undocumented immigrants face fears of “immigration enforcement policies, mistrust people in authority, and report facing discrimination that limits their access to essential health and social service” (Sahay et al., 2016). Undocumented parents encounter significant barriers when attempting to enrol their children in the public school system, regardless of the children’s immigrant status. The North Carolina ACLU found that “before they even show up to register [in school], many of these children or their family members face traumatic migration experiences, and many struggle with language barriers as well as economic and legal instability” (Grossman, 2019). As a result, access to primary and secondary school education, which is compulsory in the U.S., regardless of the children’s immigration status, is undermined in North Carolina.

In sum, systemic barriers to education, such as the ones examined above, are an impediment to all forms of educational attainment. Education success in K-12 school and postsecondary education is a critical factor in creating a pathway to effective acculturation and assimilation for immigrant children and youth. Their undocumented or uncertain legal status, which here may be considered a “master status” (Gonzales, 2011) above all other various social identities they may possess, is mediated by exclusionary immigration policies resulting from the practices of criminalization of immigrant families.

### Health and well-being impacts of existing barriers immigrant families face

Definitions and standards of health and well-being have long been considered historically and internationally in relation to children and their families. The World Health Organization defines health as “a state of complete physical, mental and social well-being and not merely the absence of disease or infirmity” (Organization, 1947, p. 1). The Ottawa Charter for Health Promotion conceptualized health as the “resource for everyday life” and confirmed that “to reach a state of complete physical, mental and social well-being, an individual or group must be able to identify and to realize aspirations, to satisfy needs, and to change or cope with the environment” (World Health Organization, 1986, p. 1). The Jakarta Declaration of 1997 considered health “as a human right that helps people to lead socially and economically productive lives, and above all, poverty as the greatest danger to health” (as summarized in Kristén et al., 2015). As it has been characterized, the “immigration paradox” is that even when their “families are among the poorest, least educated, least insured, and least able to access health care …, these children demonstrate better-than-expected health status, … suggesting that cultural health behaviours among immigrant families might be protective in some areas of health” (Mendoza, 2009, p. 187). Thus, research examining the health and lifestyle (e.g., their well-being) of this young population must identify the “barriers and facilitators connected with the perceptions related to personal, social, environmental, and policy or program factors” (Kristén et al., 2015, p. 2). These propositions guided the authors in analysing the full impact of immigration policies criminalizing immigrant children and their families in the U.S. and affecting children’s health and wellbeing.

Historically, immigrant children often flee their home countries to escape violence, poverty, and corruption at the suggestion of their parents or caregivers (Collier, 2015). Although some come unaccompanied, many travel with their siblings and parents, traversing miles of difficult terrain and encountering numerous difficulties along the way (Collier, 2015). Amuedo-Dorantes and Lopez (2017) found that immigration enforcement can adversely affect the children of undocumented parents. However, the scope and impact of this enforcement differs depending on the age and context of the immigrant children and youth:

Intensified enforcement raises the probability of repeating a grade for children ages 6–13 by 14%, and the likelihood of dropping out of school for youth ages 14–17 by 18%. Furthermore, younger children are more responsive to police-based enforcement, whereas older youth are more responsive to employment-based enforcement. (Amuedo-Dorantes & Lopez, 2017, p. 120)

As a result, immigration policies impose “hidden educational costs” on children, many of whom are U.S. citizens or DACA recipients (Amuedo-Dorantes & Lopez, 2017, p. 120). Thus, the criminalization of migrant parents undermines the educational opportunities and health outcomes of children, regardless of the children’s immigration status.

One major determinant of health and well-being among undocumented children, adolescents, and youth is the traumatic experiences they and their parents tend to have because of the anxiety-inducing uncertainty of their future. In fact, many immigrant parents instil a sense of mistrust in their children stemming from their daily fears of deportation that threaten to result in family separation (Enriquez, 2015). In an effort to maintain their survival, parents and children alike develop coping strategies like hypervigilance, avoidance, and distrust that relegate them to the confines of their own homes and communities (Mapp & Hornung, 2016). Research suggests that many undocumented students refrain from going to college or even drop out of high school as a result of the high levels of stress and anxiety they experience due to the anti-immigrant sentiment perpetuated by the current political climate (Amuedo-Dorantes & Lopez, 2015; Mapp & Hornung, 2016).

Coupled with language and cultural barriers, many children may find this method of social scaffolding difficult to engage in, especially in an educational system ill-prepared to help them with their educational adjustment and advancement. Furthermore, parental desire to improve children’s education and overall well-being is highly influenced by the Trump administration’s restrictive immigration policies and administrative practices that increasingly justify family separation as a way of punishing entry into the country and deterring future migration. For an extensive discussion on historical shift towards criminalization of immigrants through mass incarceration of undocumented Latinos and the expansion of the prison-immigration industrial complex, see Ackerman and Furman (2014).

The persistent criminalization of migrant children and their families augments the effects of the more adverse immigration policies and practices endured under the current administration. These dynamics of unjustly delegated power and disproportionate control over immigrants create a vicious cycle of despair and uncertainty for this large segment of the U.S. population. The fear of deportation has perpetually shadowed the lives of migrant children; however, this fear has significantly increased since the Trump administration’s prioritization of border security (De Vogue et al., 2017). This political shift is characterized by persistent funding requests for a Southern border wall, the indefinite detention of immigrants and their children, and the practice of family separation as a migration deterrent policy. For an in-depth analysis of the forced separation of children from their families during the 2014 and 2018 humanitarian and human rights crises at the southwestern U.S. border, see Gendle and Monico (2017), Monico, Rotabi, and Lee (2019), and Monico, Rotabi, Lee, and Vissing (2019).

### Multi-layer factors influencing the well-being of immigrant children, adolescents and youth

Protective factors are critical in counteracting risk factors, particularly among immigrant children. Research regarding the well-being of this population suggests that health developments and successful adjustments to society are mediated by various factors. They include: “(1) the assets and resources they bring from their country of origin, (2) how they are officially categorized and treated by federal, state, and local governments, (3) the social and economic circumstances and cultural environment in which they reside in the USA, and (4) the treatment they receive from other individuals and from health and social institutions in the receiving community” (Institute of Medicine and National Research Council, 1998, p. 3).

The individuals of the 1.5 generation are not considered first-generation immigrants (migrating adults) or second-generation citizens (born in the U.S.) who are not undocumented or need DACA (Portes & Rumbaut, 1996). Instead, they fall in between, migrating to the States as young children with their parents and often living with younger siblings born and raised in the U.S. Optimal assimilation of the child migrants of the 1.5 generation forms positive trajectories that are often threatened by their legal status and other structural barriers; that is, “If citizenship is denied migrants or their offspring then it seems unlikely that full economic, social or cultural incorporation would follow” (Glick & Park, 2016, p. 519). Yet, barriers the parents of this population face may not necessarily turn into a denial of opportunities for the new generations as “young immigrants or children of immigrants may benefit by cultivating their ethnic ties in their ethnic communities to develop forms of behavior likely to break the cycle of disadvantage and to lead to upward mobility” (Zhou, 1997, p. 91). In fact, research shows they can still accumulate significant social capital wherever they settle (Glick & Park, 2016).

Consequently, the provisional legal status of the DACA program allows 1.5 generation young adults access to opportunities to build their social capital through higher education and work opportunities. Sometimes unaware of their immigration status, undocumented high school graduates that become eligible to attend universities and colleges in the U.S. are often confronted with an uncertain future (Lopez, 2010); nearly 100,000 of them are estimated to graduate annually (Zong & Batalova, 2019). These young students face numerous obstacles before attaining a higher education deterring many from not aspiring to it (Colotl, 2017). From a lifespan perspective, the “American Dream” dominates the childhood of DACA beneficiaries while involving great challenges “to achieve the promise of a college education. In 16 states, DACA students are either prevented from attending their in-state public college or university, or are forced to pay prohibitively expensive out-of-state tuition to attend their home-state public institutions.” (Núñez, 2017, p. 2).

Identifying and deciphering the emotional landscape undocumented children and youth experience endure throughout their lives offers researchers a new way of interpreting the prolonged uncertainty of undocumented life trajectories or their ambiguous loss. Ambiguous loss is a form of grief that develops in environments of uncertainty, blocking cognition, freezing emotions, and severely hampering individual and family functioning (Boss, 2010). These negative emotions can lead to depression, rejection, and insecurity, including high levels of fear and anxiety (Pérez et al., 2010). This trauma manifests in their lack of social support systems and mistrust of authority figures, preventing them from accessing mental health resources and campus services (Pérez et al., 2010).

Immigrant students’ apprehension about university support systems compels them to use alternative means of coping with the impairing circumstances. These alternatives will determine the depth of their resiliency or ability to rebound from adversity (Abrams, 2001). As trauma is magnified and compounded throughout the unspoken experiences of these immigrant students and their families, their mental, emotional, and psychological health further deteriorates. Professionals in higher education often notice that these students constantly find themselves grappling with “feelings of shame, trepidation, anger, despair, marginalization, and uncertainty” (Pérez et al., 2010, p. 37).

The health and well-being among migrant children and their families can be better understood through intersectional perspectives, multi-level systems, and biopsychosocial dimensions. A cross-group comparative study of acculturative stress related to family and other social contexts among 416 documented and undocumented Mexican and Central American immigrants living in two major cities in Texas found that restrictive immigration legislation enacted in 1996 produced “higher levels of the immigration challenges of separation from family, traditionality, and language difficulties than documented immigrants, [and] both groups reported similar levels of fear of deportation” (Arbona et al., 2010, p. 363). In fact, Olivos and Mendoza (2010) found a convergence of “language proficiency, socioeconomic status, immigration status, and race/ethnicity … [that creates vulnerability, restricting] parental and community engagement … as consequences of social inequities which remain unaddressed in the institutional context of public education” (p. 339). Indeed, more restrictive immigration policies produce higher levels of stress among immigrant children and their families.

Based on their differences from the white, privileged culture that prevails in the U.S., migrant children and their families arrive to a “colonized context” that imposes a “second border” while they begin a life in “Spanglish” and in an “internally colonized community” where racism prevails (Castro-Salazar & Bagley, 2010; Morales, 2002). In fact, their immigrant status is often compounded by “multisubjectivity” and intersecting “identities such as sexuality, race, culture, socioeconomic status, religion, national origin, language, and immigration status” (Morales, 2002, p. 51); in this way, they “figure” (or take agency of themselves and their community) directly affecting their health and well-being.

A meta-analytical review of an extensive dataset of studies on immigrants during adolescence offers important findings regarding the health impact of systematic discrimination.

Greater perceptions of racial/ethnic discrimination were linked to more depressive and internalized symptoms; greater psychological distress; poorer self-esteem; lower academic achievement and engagement; less academic motivation; greater engagement in externalizing behaviors, risky sexual behavior, and substance abuse use; and more associations with deviant peers. (Benner et al., 2018, p. 855)

In sum, in response to the harmful effects of increasing immigration policy enforcement on immigrant children and their families, they have developed a wide range of compensatory coping mechanisms. Some of these coping mechanisms are effective in minimizing the negative effects of those policies and decreasing the likelihood of risky behaviour. However, as discussed later in this paper, some of these coping mechanisms may be characterized as maladaptive from a clinical perspective resulting in negative consequences and outcomes for immigrants and their families.

## Method

Life stories and in-depth interviewing are essential for understanding the dense narratives of people and their meaning making, particularly among those who “navigated across and between racialized historical, socioeconomic, political and culturally colonized boundaries, barriers and contexts” (Castro-Salazar & Bagley, 2010, p. 26). Accessing the worlds of children and childhood via the recollections of adults is logical and appropriate possibility due to the easy access many adults have to their childhood memories (Philo, 2003). As Harris and Valentine (2017) confirmed, this research approach “is not to suggest that early experiences are deterministic. Rather, individuals can reflect on their own lives and encounters and choose to change or react to wider social relations in new ways such that they produce and embody new dispositions” (p. 505). Given the relative nature of life stories, research rigour does not involve reliability (a term used in positive/post-positive methodological paradigms) but it involves confirmability or the participants’ validation of the findings (Guba & Lincoln, 1989), which in this study was done during the interview only. The interviews involved retrospective views of childhood. Although these narratives of life trajectories in adulthood have their limitations, they are key to identifying critical issues in child development, particularly for the development of strategies to reduce risks among youth and young adults later in life. This being one of the intentions of the study reported here.

To understand the participants’ agency and identity, social practice theory of self and identity was used as a framework to construct meaning of the life-span narratives (Holland et al., 1998). The study focused on the effects of the fear of deportation on the mental health of Hispanic immigrant college students and identified the salient coping mechanisms they engage throughout their life education experience. To this end, semistructured, mixed-method interviews were conducted, using both closed questionnaires and open-ended questions to engage participants in the dialogue. Thirteen, 50–70 minute interviews (equivalent to a class period) were conducted with students from a variety of citizenship statuses ranging from temporary protection to no documentation.

To understand the sample better, a participants profile is explained here. The 13 participants were all college students; 11 were DACA beneficiaries and 2 undocumented. All of them were over the age of 18, attended public or private institutions of higher education, and were recruited using gatekeepers and snowball sampling. Two of the thirteen identified as male while eleven identified as female. Additionally, two of the participants openly expressed their membership of the LGBTQIA community. Although some participants identified as Hispanic or Latinx, some claimed Mexican or Colombian heritage. All of them were bilingual, although their language proficiency in Spanish was not tested because this would have been beyond the scope of the study. All of them had arrived in the USA as minors, particularly DACA beneficiaries whom to become eligible to this immigration program they needed to demonstrate that they were brought to U.S. before their 16th birthday and prior to June 2007. Regardless of immigration status, the youngest participant was 18 and the oldest was in his early 30th. Most students revealed that they had lived with their families before coming to college and resided in North Carolina for 5 years or more. Families consisted of their parents and often their siblings with several mixed citizenship statuses. As compared to the average family income at their respective institutions, many participants reported having a lower socioeconomic status than their peers, thereby needing significant merit-based financial assistance.

Two validated surveys were used: a Fear of Deportation Instrument, adapted from Arbona et al. (2010), and a Coping Strategies Instrument, adapted from Mena et al. (1987). The modified version of these instruments served to initiate points of conversations and follow-up questions. For the purposes of this article, special attention was given to the childhood life-narratives of the research participants. Additionally, the authors held informal meetings with four immigrant service providers to gain further insight on the challenges they have encountered since the beginning of the Trump administration.

**Figure 1.**
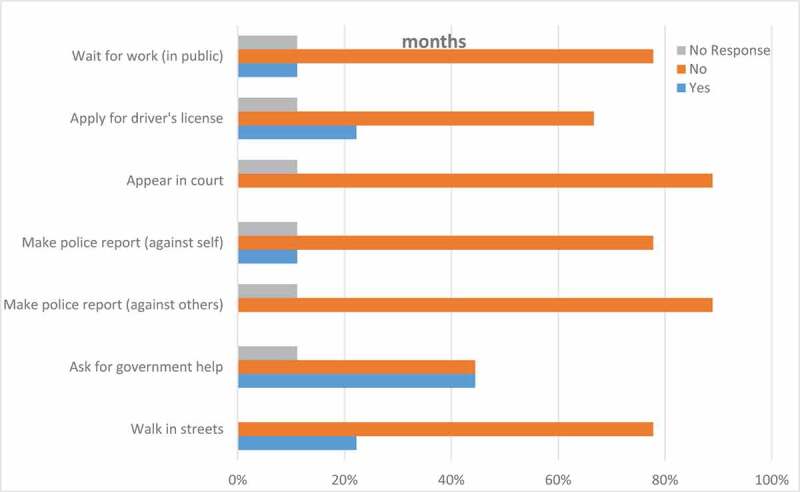
Fear of deportation instrument—activities avoided in past 12 months

**Figure 2.**
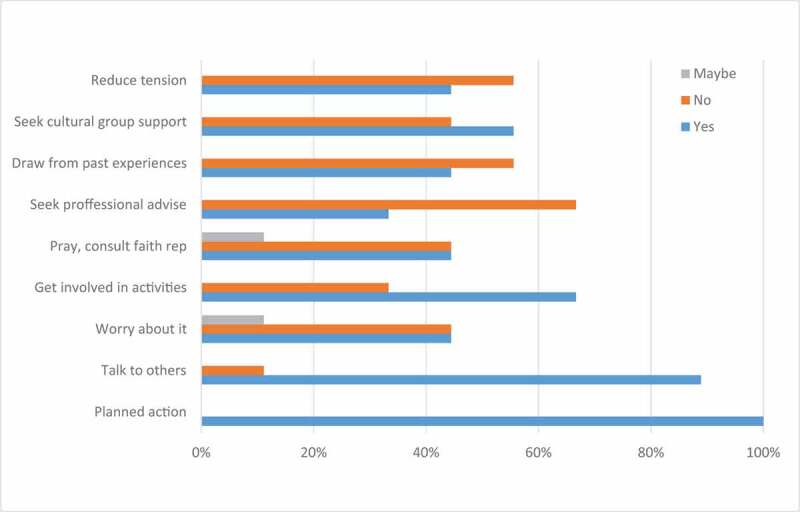
Coping strategies instrument—activities undertaken

**Figure 3.**
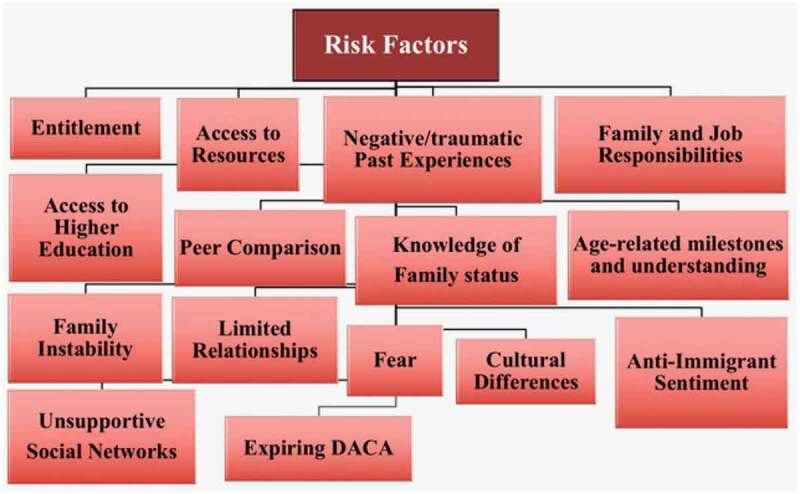
Risk factors among research participants

**Figure 4.**
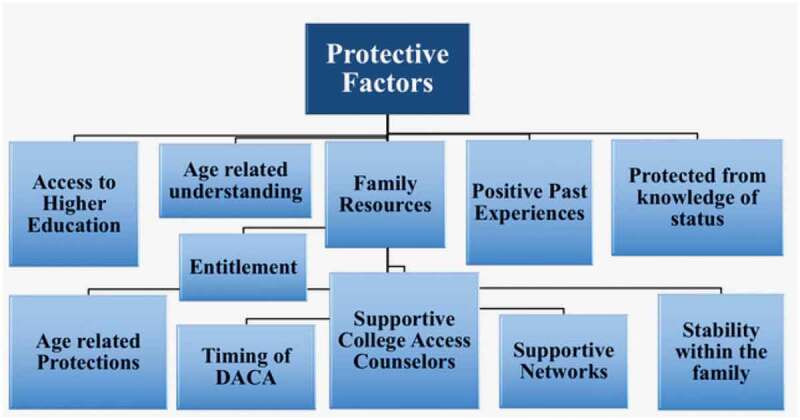
Protective factors among research participants

Interpretive qualitative research was used for data analysis, identifying themes from codes pre-identified from the literature and emerging from the interviews (Guba & Lincoln, 1989; Lincoln & Guba, 1985). Although analysis was conducted using induction by unitizing and developing memos from data collected through the interviews, deduction was used by identifying root codes selected from the literature to frame the data and begin production of a coding tree. Using codes inferred in data analysis, risk and protective factors were identified (see Figures 1 and 2). Dedoose was used for qualitative analysis of the interview transcripts. Codes related to the participants’ childhood experiences were identified and used to capture participants’ quotes and analyse them for meaning-making purposes. Excel was used for analysis of the quantitative survey data (see Figures 3 and 4). Preliminary study results were presented in various conferences, to confirm findings with audiences serving the study population and to formulate more appropriate institutional next step recommendations.

## Results

After consenting to participate in the study, participants completed two quantitative surveys at the beginning of the semi-structured interview. The results in this section are presented in the order in which they were administered.

### Quantitative results

As indicated earlier, two validated measures were utilized to survey the participants before conducting the semi-structured interview; that is, after participants have consented to participate in the study. The Fear of Deportation Instrument, adapted from Arbona et al. (2010), consists of 7 activities that participants are asked to indicate if they may or may not have avoided in the past 12 months due to a fear or concern that you may be at risk of being deported. As a follow-up question in the same survey, participants are asked to explain under which circumstances they participated or not participate in each of the 7 listed activities. The Coping Strategies Instrument, adapted from Mena et al. (1987), inquired about how frequently participants cope with anxiety during times of high stress and emotional pressure. It listed 9 common coping strategies that participants identified as described behaviours or not. The instrument also asked participants to provide an example of the given strategy they had done before.

Figure 1 depicts the results of the Fear of Deportation Instrument. We found that the activity students indicated that had done the least in the last 12 months was to ask the government for help, with six of the 13 participants avoiding it. Only three participants feared walking in the streets and applying for a driver’s licence. Further, only two reported being afraid to file a report to the police for an infraction committed against one’s person or feeling fear while waiting on the street corner to get work.

Figure 2 displays the results of the Coping Strategies Instrument. All of the participants listed that they utilized pursuit of knowledge about a particular problem and taking a positive, planned action as a form of coping. Data also indicates that almost all (twelve of the thirteen) participants talked with relatives and friends about the problems they faced, nine chose to get involved in other activities in order to avoid persistently thinking about the problem, and seven sought support from members of their own cultural group, in any way they defined it. In addition, six of the participants indicated that: they were worried about the problem, prayed or consulted with a faith figure, drew from past personal experiences, or tried to reduce tension through various means. Only four selected that they sought professional advice from a physician, psychologist, or counsellor.

### Qualitative results

To interpret and analyse the data through a more intersectional and reciprocal lens, we focused on using the framework of risk and protective factors—see Figures 3 and 4. These factors were found to be present within the individual, family, and community environments as highlighted in the following sections. In the individual level, participants reported how their social identities further problematized the consequences of being undocumented or in a temporary status. These concerns radiated out into their family contexts as fear of parental deportation weighed heavily on their decisions to pursue higher education, career choices, and even their decision to pursue government-related programs such as DACA and obtaining a driver’s licence. Finally, at the community level, the support the participants accessed became an influencing factor in their lifespan, from childhood into adulthood.

### Illegality and uncertain statuses as a master status among immigrant children and youth

One participant remember the immigrant journey to the U.S. since childhood but did not know that being undocumented was a major influence while growing up. No student interviewed was resentful towards their parents for their lack of disclosure regarding their illegality. Only one participant questioned why the parental figures brought them to the U.S. Most students were supportive of their parents’ decisions to immigrate and bring them along. They did not question their parents’ decisions not to tell them about their undocumented status. While growing up, most participants felt different because of their family socio-economic status and ethnicity. Their college aspirations and interface with immigration policies became the catalyst for them to realize their lack of documentation.

Immigrant service providers report that since the beginning of the Trump administration immigration has adopted more restrictive policies and the police increased its law enforcement among those who appear to be immigrants. Even if undocumented students endure high levels of stress and anxiety regarding law enforcement, fear of deportation was not always identified as the explicit cause. For example, a requirement for obtaining a driver’s licence in North Carolina is to be accompanied to the Department of Motor Vehicles by a valid adult licence holder, a commodity many undocumented adolescents do not have. Multiple students revealed that the only reason they were able to receive their licence, a privilege that alleviated the load of driving from their parents, was because of the help of a community member who was willing to accompany them to the testing centre. In this study, most students talked about how they avoided or delayed having a driver’s licence at the age of 16 years old. They justified it with not being ready to do so to appear normal with respect to their peers who were getting their licence permit.

And then I came here [the U.S.], and you know, I didn’t really know about my legal status until it came down to applying for scholarships and then realizing that like I couldn’t get it because I wasn’t a U.S. citizen. (E1P2. 1/26/18)

As indicated earlier, the score in the Fear of Deportation Instrument was surprisingly low for this small sample; however, the follow-up questions made to this instrument offered some insights as to how this population may experience fear. For instance, during the interviews, students reported feelings of stress and anxiety caused by circumstances related to their status, manifested in disrupted romantic relationships, inability to study abroad, unbalanced work or family responsibilities, and limited career and internship opportunities.

The immigrant identity of the interviewed youth included being a first-generation of immigrant (born in another country), Hispanic (regardless of whether the person speaks Spanish or other languages), Latinx (including those self-identified as LGBTQIA+ individuals), or with specific heritage, such as Mexican or Colombian. However, study participants described not knowing of their undocumented status (“master status” or the position in a social structure) until they were seeking scholarships to attend college. One participant confirmed the dominance of illegality or uncertain legal status during the transition from childhood to adulthood, particularly in the deeply rooted fear of racial profiling by members of law enforcement, which triggered a persistent fear of federal and state government entities and employees.

I think it’s … well when I was younger, I know I don’t think it was necessarily my family’s fault but I always saw the police and people from the government as like bad people. So, I think that stayed with me and when I fully understood my status of undocumented it became more of a fear for my family. (RV3. 7/21/18).

Being an immigrant intersected with other salient identities such as their race and gender. In fact, a participant described experiencing internalized oppression through their multiple social identities as they confronted adverse immigration policy and public perceptions.

Experience[ing] like little things like that where I knew like it wasn’t already not okay to look Hispanic but then like if they really knew that I wasn’t born here, then that adds on more: like what would they think about me or what would they say about me? (E1P1 12/18/17)

Gender and racial identity intersected with the immigration status of the participants. Female participants indicated how their identities as women and immigrants manifested in multiple forms: fear of walking in the streets at night, of going to unfamiliar places, and of interacting with others different than them, particularly if those individuals display anti-immigrant sentiments.

I think that as a female it’s [fear of walking in the streets] something that you experience. I don’t think I’m the only person, but knowing that I’m undocumented and even though I do have DACA, having that extra fear of like just coming across people whose ideas are really negative toward me. (E1P1. 12/18/17)

### Barriers to navigating education from childhood into adulthood

Most of the students claimed that their guidance counsellor was the main source of information for overcoming academic barriers; they helped them to overcome the obstacles posed to undocumented immigrants because they believed in them. This is because were not eligible for FAFSA and did not know where to go; academic counsellors provided the support they didn’t have at home. Legal documentation was not the only barrier that these undocumented immigrants encountered. One participant in primary school often hear anti-immigrant sentiments and experienced bullying by her U.S. citizen peers. Without regard of their legal status, students were aware that they were different because they were immigrants, and of their Hispanic origin.

In college, a lot of barriers to immigrant youth were related to their socioeconomic status. Most DACAmented had undocumented parents, who lacked the financial resources to pay for college, and most parents had low paid or low skilled jobs. They thought they would probably not be going to college because their parents did not. Guidance counsellors played a mediating role for these immigrant youth to access college education. Opportunities to resources that didn’t require legal status were opened up to them. This is why the Odyssey program and other grant programs not requiring citizenship are very important in guaranteeing higher education to immigrant youth.

For some students, their different status became evident during the school field trips when they could not go on fieldtrips. Their parents wouldn’t sign the travel authorization because they were afraid for their children to go out of state, presumably because of their undocumented status. A major barrier student participants experienced in college was their inability to fully access defining collegiate experiences, such as study abroad.

I think that the first thing just because I know I had that opportunity because you could study abroad with DACA if you had the permission, if you had the evidence that you’re studying abroad. So that was my dream to study abroad, but now that was taken away. Studying abroad was a dream of mine, and now I can’t do it anymore. (S1P1. 7/9/18)

These barriers due to the master status restricted students’ ability to engage in collegiate life their U.S. citizen peers enjoyed in higher education institutions, discouraging them from seeking additional resources, including mental health care. For instance, 9 of participants indicated that they did not seek professional advice from a physician, psychologist, or counsellor.

### Adverse health impact of immigration policies on immigrant children, adolescent and youth

Participants reported a heightened fear for the anticipated ending of DACA benefits. The expectation that their current DACAmented status will be revoked produces frustration about their present lived experience and a source of anxiety and stress for their future. The uncertainty of the immigration status produces a particular form of ambiguous loss, which can be only mediated by resiliency and protective factors, as exemplified below.

[The] Trump administration, he tried to get rid of DACA. So, what happens when my DACA expires and I can’t renew it, you know? I’ll have a degree, but I’m not going to be able to use that degree because I don’t have a permit to work in the USA. So, it kind of feels like I’m wasting my time, but I’m not because an education is something that they can never take away from you. (S1P1. 7/9/18)

Some students rejected the notion that their opportunities were totally taken away from them because they approached their DACAmented status with pessimistic perspective and lowered expectations. In this instance, mistrust of law enforcement and belief in an unreliable government proved to protect them from the consequences of DACA’s rescission.

I knew I was never a U.S. citizen, so I never felt like I lost that. But just with DACA, I got DACA and then in a way, it was taken away from me. So, I don’t know; it’s just my protection that I lost. I don’t know. It’s just with police I don’t think I will ever feel safe. (E1P2. 1/26/18)

The state of uncertainty can undercut the motivation and drive undocumented youth have to succeed in life. In turn, their hope for a better future for themselves and their families, and their desire to belong are challenged. Without the promise of education, the aspiration for social mobilization weakens and their social agency undercut at a point of critical life transition.

### Family and community support as a multi-impact factor of immigrant’s lifespan

Combined with the roles of the individual and the family, the community in which the study participants are immersed proved to be extremely vital to their success. Without the support and resources of an active community, students interviewed are likely to have succumbed to the legal and systemic barriers that prevented them from accessing an array of resources. Most research participants credited the influence of their high school guidance counsellors as the main reason why they decided to pursue higher education seriously.

My high school was blessed with our college advisor. She was—she knew like there were students with this situation and she was like, “Hey this is what you got to do. You can go to school. You can go to school. You can go to school.” She was like, “Don’t let nobody tell you that you cannot go to school.” (G1P1. 1/26/18)

For the study participants, family becomes both a source of stress as well as a source of protection. The stress of being undocumented affects an individual’s life in significant ways; however, many participants reported that the weight of their families’ immigration status can affect them even more. Despite their own protection through the DACA program, students indicated that when their parents are not protected and are still at risk of being detained at any moment, high levels of stress and anxiety compel them to choose colleges closer to home in support of their parents. Students communicated both an explicitly and implicitly felt obligation to their families to select career paths that were financially lucrative enough to support both themselves and their families.

The fear of the parent’s detainment leaves their children in a state of constant fear, not only for themselves, but also for their families. After all, parents drive, work, and live under the daily threat of deportation because they are undocumented. One student even expressed apprehension towards applying for DACA due to the threat of releasing such information would have on her entire family.

Because my family, they’re not bad people. And they, my family, deserve the world and I don’t want them to be in danger, that’s why also initially I didn’t want to apply for DACA because I didn’t, they have all the information on me and if it was just me, I wouldn’t care because it’s just me … They have my information they have a way to get to my family and that’s terrifying because they’ve already gone through so much. (RV3. 7/21/18

Data analysis identified several protective factors, like effective guidance counselling and supportive social systems, which could offset some of the negative outcomes in the nested community systems. Students reported that knowledgeable and socially aware friendships helped counterbalance potentially volatile situations as citizen community members leveraged their privileges to help them. Mainstream media often incorrectly refer to this community support network as an effort to provide “sanctuary” for undocumented immigrants, who would otherwise face Immigration and Customs Enforcement (ICE) detention, and subsequently, imminent deportation. This false characterization fails to consider the interdependent nature of immigrant communities and their surrounding areas. The need undocumented immigrants and their citizen neighbours have for one another is founded in the idea that everyone deserves to feel safe in their communities, a belief that proved exceedingly protective for students and corresponding families The statement from a participant below exemplifies the imperative of social connections and concrete familial and community support when confronting adversity and ambiguous loss.

I remember one morning I didn’t get to school ‘cause someone called ICE on one of our neighbors and my friend, she is documented and she immediately texted me, she told me, “Hey don’t walk out of your house, just stay inside.” (RV3. 7/21/18)

In addition to community awareness, resource knowledge that comes with active engagement between undocumented youth and their supporters helps address some access barriers that may exist for undocumented immigrant students. Being a first-generation college student in the U.S. presents a multitude of academic barriers that more privileged students never have to encounter, like the understanding and value that effective college preparation has on the undergraduate admissions process. Extracurricular activities, a strong GPA, and robust recommendation letters all contribute to a compelling college application; however, many immigrant students whose parents never attended school in the U.S. may never receive this insight.

I had incredible people in my life who told me like “these things [extracurricular activities] are important. And when you get to senior year, colleges will want you to be well rounded.” Something that I would have never known from just myself or my parents or anyone in my life, in that part of my life. (A2. 8/2/18)

Connections with knowledgeable support systems helped students to access this important information, preparing them better for the higher education admissions process and college experience.

## Discussion

Unlike quantitative studies, qualitative studies produce results that are not generalizable; thus, they must be interpreted in that context and any attempt to make generalizations from the results denies the nature of interpretive research. Yet, the findings are of sufficient interest and relevance to the growing literature on how young immigrants and children of immigrants are growing up in the U.S. Given the small sample size, the quantitative data collected was examined in combination with the qualitative data gathered through the interviews. An additional review of the literature on the issues raised by the participants became essential to understand the results.

The scores on the Fear of Deportation instrument were surprisingly low for all items considered while the scores in the Coping Strategies Instrument were expectantly high, except for the item on professional advice, which was expectantly low. The false sense of security that immigrant children, adolescents and youth with undocumented status tend to experience prior to their transition from childhoold into adulthood (Gonzales & Vargas, 2015) may explain the results. In fact, the study participants reported low scores on the Fear of Deportation measures while still reporting experiences of uncertainty, and possible trauma and ambiguous loss, as well as apprehension in using support systems, and even maladaptive behaviours. Since both instruments were used to initiate points of conversations and follow-up questions, the participants’ narratives tend to offer greater insights on how their coping mechanisms mediate their fear of deportation.

The study suggests that the DACA program has resulted in mixed outcomes for the well-being of the interviewed recipients’ transitioning from childhood to adulthood. Relevant systematic research concluded that “While DACA has shifted the experiences of undocumented young people in some ways for better, the transition to adulthood is still significantly shaped by their undocumented status” (Gonzales & Burciaga, 2018, p. 158). The condition of “illegality” influences the experience of DACA recipients as individuals with respect to their nation-state but also captures their experience in the sociopolitical and cultural realms (Gonzales & Burciaga, 2018). The life trajectories of DACA students are complicated by the different regulations at the state and county levels regarding access to education in higher education; thus, regardless of whether they go to college or seek employment, the “transition to illegality” is not only influenced by their school experience in childhood but also by their place of residence (Gonzales & Burciaga, 2018).

Using the risk and protective factors as a theoretical framework in this study has been useful in understanding that “being undocumented is a risk factor for mental and physical health conditions” (Enriquez et al., 2018, p. 193). Qualitative data revealed that many undocumented immigrants possess a combination of marginalized identities that compound and complicate the risk factors associated with their undocumented identity. The fear of police presents an authentic and immediate threat for immigrants with darker skin tones as law enforcement using racial profiling often targets and prosecutes undocumented immigrants. Quantitative data suggests that even when participants did not seem to have avoided the activities listed in the Fear of Deportation instrument, asking the police for help was the least action done. The fear of deportation among the study participants seemed to depend upon their personality traits (some displaying more confident than others) and mental health state (some reporting depression and anxiety more than others). Most participants reported no fear of deportation for themselves but for their parents, presumably experiencing fear of family dissolution as a result of deportation of their parents.

Immigrants are more afraid for their lives than ever before under the Trump administration, despite the knowledge that President Barack Obama deported more people than any other U.S. president. In fact, since Trump has taken office, deportations have dropped to its lowest since before the Obama administration began (Vaughn, 2017). Nevertheless, fear among immigrant communities continues to grow since 2016 as the combination of antagonistic anti-immigrant rhetoric and targeting of noncriminal immigrants has instilled a deeper belief that those who were once “safe” are now at risk (Colotl, 2017).

The study found that many participants have experienced deep emotional trauma since their arrival to the U.S., beginning during their childhood years. Yet, this trauma is sustained across multiple socio-ecological systems as individuals and their interrelated identities interact with their proximate and distal contexts (Bronfenbrenner & Morris, 2006). The quantitative data indicates that participants use multiple mechanisms for coping, including planning their actions, talking to others, getting involved in extracurricular activities, and seeking support from their cultural groups. Most important, for the most part, participants did not seek professional advice. Thus, it is understandable that in some cases, their coping mechanisms would have evolved into maladaptive behaviours, such as driving without a valid licence.

To resist the external threats, undocumented migrants develop resiliency through counter narratives and building personal and community capacities to endure adversity. Muñoz (2015) articulates well the emergence of counter narratives to the legal challenges they face while constructing their own identity as Latinx: “youth have strategically constructed their own compelling messages, highlighting not only their pride in being undocumented, *unafraid* and unapologetic, but also how this adopted identity informs and shapes their other social identities” (italics added for emphasis, p. 7). This discourse of being unafraid cannot be understood in the context of the family and community support, in the way in which coping mechanisms and protective factors mediate risk factors, to make these immigrant youth into more resilient beings.

This study suggest that building resilience is a key ingredient for rebounding against adversity, particularly among immigrant and undocumented children and youth. This implies that “resilience is neither inherited nor stable, and can be cultivated in children, thus enhancing the likelihood they will overcome challenges” (Calhoun et al., 2018, p. 1). Vesely et al. (2017) offer a community-centred model that takes into consideration “uncertainty and family separation; ecosystemic trauma and stress, including institutional betrayal trauma; limited navigational capital; and erosion of collectivism and community solidarity” (p. 120). In other words, it is necessary to take into account all of the potentially adverse factors to migrant’s well-being in order to develop protections that enhance their resiliency.

Secondary education settings provide an opportunity to service providers and guidance counsellors to make a significant impact in the lives of undocumented youth. By law and job description, school counsellors are required to provide effective and comprehensive services to all of their students, including those who may be undocumented, but to do this, they must be aware of all of the resources that exist on behalf of immigrant students (Morrison et al., 2016). In addition, school counsellors must be competent in a number of areas, such as career services, college access, and mental health resources. Each of these services must be nuanced and tailored to fit the requirements and needs of undocumented students. This adjustment takes time, effort, adequate training, and resources. For low-income individuals and schools receiving low levels of resource allocation and attention, this may be a nearly impossible task.

Condon et al. (2016) make the case for the adoption of a policy inclusion effects (PIE) framework that enables immigrant inclusion in existing safety nets to produce better educational attainment. Thus, inclusive programs must involve “integrity, transparency, and solidarity, [and] mapping out a theoretical framework grounded in a trauma-informed, anti-oppressive, and intersectional approach” (Soberano et al., 2018, p. 22) in order to become more responsive to the mental health needs of migrant children and youth. These approaches are critical in public schools as well as college-level public and private institutions.

From a lifespan perspective, colleges and universities with policies and practices that consider welcoming students from vulnerable populations, regardless of their immigration status, offer hope to immigrant families while bringing about social justice. To the approximately 25,000 DACA recipients residing in North Carolina, the Senate Bill 615 is a concrete form of community support. If approved, this legislation would grant “in-state tuition at UNC [University of North Carolina] constituent institutions and state community colleges if they meet two requirements: they received a high school diploma or equivalent diploma within North Carolina, and they attended in-state schools for a minimum of two consecutive years immediately prior to completing high school” (Lesnewski, 2019, p. 1). As of November 2019, this bill passed the first reading in the Senate. North Carolina is among one of the five U.S. states that have enacted legislation to prohibit either in-state tuition rates or attendance at public postsecondary institutions for undocumented students; however, favourable legislation, similar to the proposed bill in this state, has been adopted in at least 18 other states, expanding life opportunities to immigrant youth seeking higher education (National Conference on State Legislatures, 2019).

North Carolina’s policies towards immigration have not always been welcoming since many of its counties have a history of increased immigration enforcement. Between 2007 and 2016, North Carolina’s 287(g) program, a voluntary immigration enforcement agreement between a local sheriff’s office and ICE, was the nation’s leader in counties with active agreements (Armus, 2019). Studies on the impact of 287(g) agreements indicate increased mistrust and hostility between the immigrant community and law enforcement creating a chilling effect on cooperation and reporting of crime (Hermann, 2018). Currently, only 4 counties in North Carolina still have 287(g) agreements; however, the state legislature has enacted several measures to prevent undocumented students from accessing higher education by requiring them to graduate from North Carolina high schools and withholding in-state tuition (uLEAD (University Leaders for Educational Access and Diversity) Network, 2019). ICE officials have committed to non-enforcement in “sensitive locations” preventing them from “performing arrests, interviews, searches and surveillance at schools, hospitals and churches” (Molina, 2018). Nevertheless, this policy does not prevent ICE agents from conducting checkpoints and raids near college campuses and dorms further increasing immigrant student’s fear while attending college (Molina, 2018).

## Conclusion

Current immigration policies have been found to reinforce the “master status” of being undocumented or of uncertain status among children, adolescents, and youth born outside of the U.S. territories while also affecting their human and social development. Since enacted, DACA has provided some with an opportunity to live without fear of deportation as long as their temporary status is extended. DACA created an incentive for graduation among youth with uncertain immigration status, a growing population in the U.S. As reported by study participants, the reversal of the DACA program and the resulting threat of deportation has exacerbated their ambiguous loss and uncertainty young-age immigrants experience culminating in a corresponding health impact in the forms of stress and anxiety.

Among study participants, the prolonged grief developed in environments of uncertainty results in a growing inability to plan for the future and make meaningful relationships. The immigrant family mentality of caution and seclusion can prove extremely detrimental to children looking to succeed in a network-based world. Next to education, social connections are considered highly influential in providing opportunities for success in the labour market. Thus, the revocation of DACA and criminalization of young-age immigrants and their families is only likely to narrow their chances to succeed in a “land of opportunities” that the U.S. claims to be.

North Carolina is one of several states where Dreamers are locked out of college educational opportunities through inaccessible in-state tuition (Núñez, 2017). Private universities are often too expensive and have limited scholarships for DACA recipients and undocumented students, as those interviewed claimed. As confirmed in this study, prior to college, many high school students see their educational opportunities undermined by policies at the state and federal levels, all of which discriminate against young age immigrant, particularly those who are undocumented or with uncertain status. Favourable legislation, including that granting in-state tuition to DACA recipients and other undocumented youth, would enable them to pursue their childhood dreams.

The study findings, selectively reported in this article, identified a number of risk factors that these Dreamers experienced during childhood, as well as the critical role protective factors played in mediating their fear of deportation. Participants reported experiencing deep emotional trauma since their arrival in the U.S., beginning in their childhood years, and in some cases developed behaviours considered maladaptive, adversely affecting their health and well-being through their lifespan. Their narratives suggest that in their transition from childhood to adulthood, they experienced a turning point characterized by the dominance of illegality and uncertain status as their new “master status.” Yet, the coping mechanisms they used throughout their lifespan seemed to have minimized the adverse effects. More favourable immigration policies and enforcement practices would likely reduce risks and increase the protective factors identified in this study, at least among the study participants. Community support from public and non-public institutions, at the federal, state and country levels have the potential to address many of the challenges DACA recipients and other undocumented children, adolescents and youth face under the current U.S. government.
